# Dielectrically-Loaded Cylindrical Resonator-Based Wireless Passive High-Temperature Sensor

**DOI:** 10.3390/s16122037

**Published:** 2016-12-01

**Authors:** Jijun Xiong, Guozhu Wu, Qiulin Tan, Tanyong Wei, Dezhi Wu, Sanmin Shen, Helei Dong, Wendong Zhang

**Affiliations:** 1 Key Laboratory of Instrumentation Science & Dynamic Measurement, Ministry of Education, North University of China, Taiyuan 030051, China; pillar921@163.com (G.W.); weity1989@163.com (T.W.); shensanming@nuc.edu.cn (S.S); donghelei@nuc.edu.cn (H.D.); wdzhang@sxedu.gov.cn (W.Z.); 2 Science and Technology on Electronic Test and Measurement Laboratory, North University of China, Taiyuan 030051, China; 3 Department of Mechanical & Electrical Engineering, Xiamen University, Xiamen 361005, China; wdz@xmu.edu.cn

**Keywords:** temperature sensing, dielectric resonator, relative permittivity, high-temperature environment

## Abstract

The temperature sensor presented in this paper is based on a microwave dielectric resonator, which uses alumina ceramic as a substrate to survive in harsh environments. The resonant frequency of the resonator is determined by the relative permittivity of the alumina ceramic, which monotonically changes with temperature. A rectangular aperture etched on the surface of the resonator works as both an incentive and a coupling device. A broadband slot antenna fed by a coplanar waveguide is utilized as an interrogation antenna to wirelessly detect the sensor signal using a radio-frequency backscattering technique. Theoretical analysis, software simulation, and experiments verified the feasibility of this temperature-sensing system. The sensor was tested in a metal-enclosed environment, which severely interferes with the extraction of the sensor signal. Therefore, frequency-domain compensation was introduced to filter the background noise and improve the signal-to-noise ratio of the sensor signal. The extracted peak frequency was found to monotonically shift from 2.441 to 2.291 GHz when the temperature was varied from 27 to 800 °C, leading to an average absolute sensitivity of 0.19 MHz/°C.

## 1. Introduction

Instrumentation is a key generic technology in the gas turbine industry that influences the development cost, efficiency, and competitiveness of gas turbine products. The very hostile gas turbine environment presents unique challenges for instrumentation. The demand for greater efficiency is steadily increasing the temperature and pressure in engines [1,2,3]. The gas turbine output power is increased by 10%, and the efficiency is increased by 1% for every 55.5 °C increase in temperature. The European Virtual Institute for Gas Turbine Instrumentation identified three areas where the lack of adequate instrumentation capability is perceived to be either holding back gas turbine engine development or leading to increased uncertainty in design methods and component life prediction [4]: measurement of gas temperature, pressure, flow, and blade tip clearances at very high temperatures (>1000 °C); measurement of component temperatures in the hottest parts of the engine; and measurement of component vibration on very hot components.

The temperatures inside gas turbines are usually indirectly obtained using temperature sensors, such as thermocouples. By measuring the voltage of the thermocouple and amplifying the voltage signal through a series of amplifier devices, the temperature can be obtained. Nevertheless, thermocouples suffer from the disadvantage of inaccuracy and cannot perform in situ temperature parameter measurements.

Sensors that work inside an engine must survive high temperatures and corrosive substances while being fully enclosed in metal. Active devices are normally unavailable in high-temperature environments because thermo-mechanical fatigue of the package material will cause the device to fail. Traditional wire-testing approaches suffer from ohmic contact failure and unstable wire connections at high temperature [5,6]. Wireless passive sensors are promising in harsh environments because they implement battery-free and contactless measurements. Currently, a wireless passive temperature sensor based on inductive-capacitive (LC) resonance could work at 800 °C using high-temperature co-fired ceramic techniques, but the LC lumped circuit suffers from the disadvantage of having a low quality factor and cannot approach the metal material characteristics, which affects the sensing distance and applications [7,8]. Wireless surface acoustic wave temperature sensors could work up to 900 °C, but chemical instabilities in the piezoelectric substrate at high temperatures limit their use in harsh-environment applications [9,10,11].

A promising approach to realize wireless temperature measurement in a harsh environment is the microwave backscattering technique, which has been utilized to achieve multi-parameter measurements, such as temperature and strain. This temperature sensor type, reported in [12], based on a dielectric resonator antenna, could work at 370 °C with a sensitivity of 307 kHz/K, although it was conducted in an open environment. A wireless passive temperature sensor that uses an integrated cylindrical resonator/antenna could work up to 1300 °C [13]; however, it has a relatively low quality factor at high temperature, which affects the reliability and accuracy of the sensor data.

**Figure 1:**
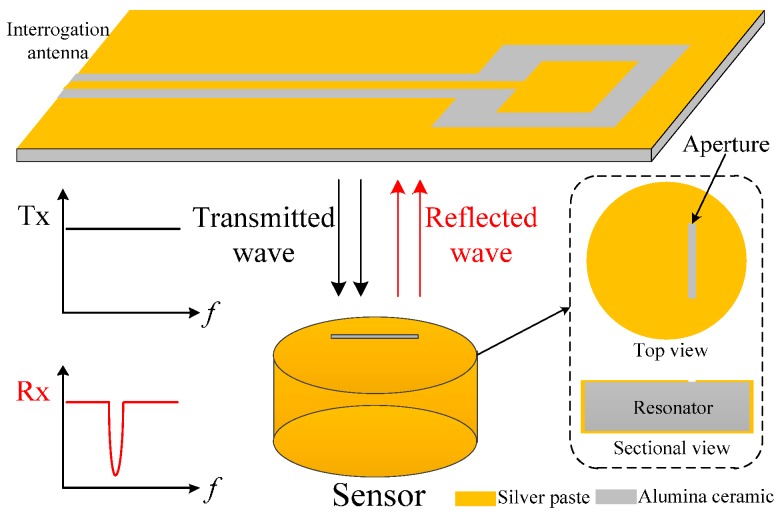
Schematic of the temperature sensor signal transmission mechanism.

The wireless passive temperature sensor presented in the present study consists of a dielectrically-loaded resonator and an aperture, which was measured at high temperature and in a metal-enclosed environment, and was demonstrated to be reliable for harsh environment applications owing to its high quality-factor stability in temperatures of up to 800 °C. The wireless signal transmission mechanism is based on radio-frequency backscattering, as shown in Figure 1. The aperture on the resonator works as a response antenna to receive the linearly-polarized wave transmitted from the interrogation antenna and coupled into the resonator. When the frequency component of the transmitted wave matches the resonant frequency of the resonator, the aperture will reflect the other frequency component back to the interrogation antenna because the matched frequency component will resonate in the resonator, which decays with time. By extracting the return loss (S_11_) of the interrogation antenna, the resonant frequency of the resonator can be obtained as a peak in the spectrum signal. This temperature-sensor type has beneficial characteristics, such as small volume, simple structure, and high quality factor, which can reduce measurement uncertainty for design validation.

## 2. Design and Fabrication of the Temperature Sensor

A dielectrically-loaded cylindrical resonator working in the *TM*_010_ mode is employed in this study owing to its stability and simplicity, as well as its concentrated area of electric fields. The resonant frequency of the resonator is determined by the relative permittivity of the alumina ceramic when the dimensions of the resonator are fixed, and it monotonically changes with increasing temperature [14]. The resonant frequency ƒ*_r_* of the resonator is expressed as [15]:

(1) $$f_{r}=\frac{c}{\sqrt{\varepsilon_{r}}\lambda_{0}}$$

where *c* is the speed of light and *ε_r_* represents the relative permittivity of the substrate. Through electromagnetic theory analysis, when the cylindrical resonator is working in the *TM*_010_ mode, the resonant wavelength *λ*_0_ could be expressed as:

(2) $$\lambda_{0}=2.62R$$

where *R* is the radius of the resonator, set to 14.5 mm. The height of resonator *h* is set to 5 mm to ensure that the *TM*_010_ mode is the dominant mode of the cylindrical resonator and to reduce the sensor size. The relative permittivity of the alumina ceramic is 9.7 at 25 °C. The resonant frequency of the resonator is calculated as 2.54 GHz according to Equations (1) and (2) and is simulated as 2.576 GHz using the ANSYS high-frequency structure simulator (Southpointe 2600 ANSYS Drive, HFSS, ANSYS, Inc., Canonsburg, PA, USA) eigenmode.

**Figure 2:**
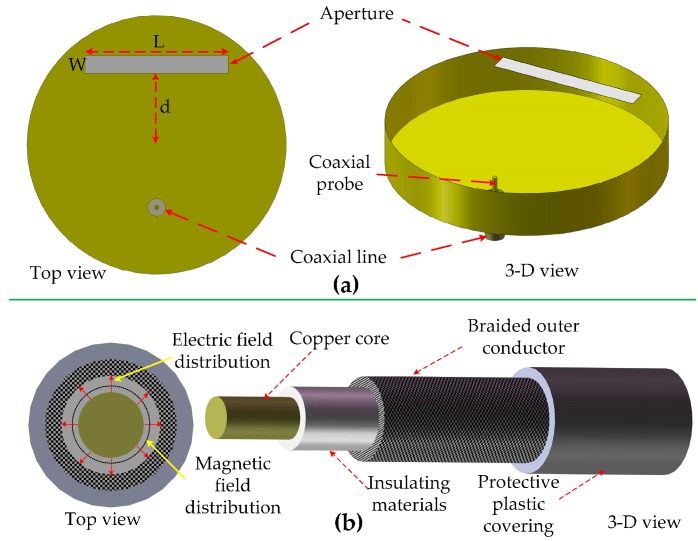
(**a**) Top and 3-D view of the integrated aperture/resonator with coaxial line; (**b**) Field distribution and configuration of the coaxial line.

The sensor consists of a resonator and an aperture. The coupling between them is essential for the signal sensing system to reduce the reflection and transmission losses. To better understand the coupling process and realize matching between the resonator and aperture, one coaxial line is weakly coupled to the resonator, as shown in Figure 2a. The coaxial probe inserted into the resonator excites the resonator, and the field distribution within a coaxial line is shown in Figure 2b. The resonator is excited through magnetic coupling because the coaxial line and the resonator *TM*_010_ mode have the same magnetic field direction.

**Figure 3:**
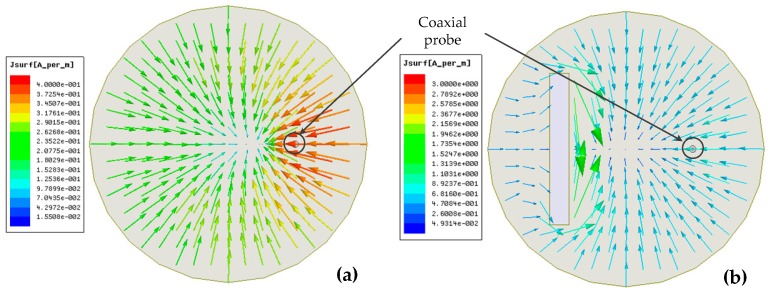
Coaxial line-feed dielectrically-loaded cylindrical resonator in the *TM*_010_ mode (**a**) without the aperture and (**b**) with the aperture.

The simulated vector current density distribution on the surface of the resonator without the aperture in HFSS is shown in Figure 3a, and the simulated resonant frequency is 2.571 GHz, which implies that the probe has little influence on the resonant frequency of the resonator. We can intuitively see that when the aperture cuts the current line, as shown in Figure 3b, the aperture will be excited, and the energy will radiate outward through the aperture.

**Figure 4:**
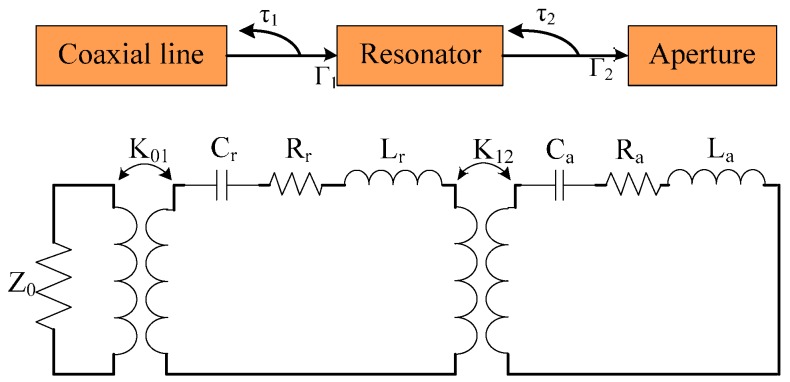
Equivalent circuit of the matching model.

The aperture works as a response antenna, whereas the resonator works as its excitation source. The equivalent matching circuit model can be roughly described, as shown in Figure 4. Reflection coefficients τ_1_ and τ_2_ represent the reflection between the coaxial line and resonator and between the resonator and aperture, respectively. When the position of the coaxial line is fixed, τ_1_ will be constant. The change in the relative position between the resonator and aperture will lead to a change in reflection coefficient τ_2_. By checking the return loss of the coaxial line, impedance matching between the resonator and aperture could be obtained.

**Figure 5:**
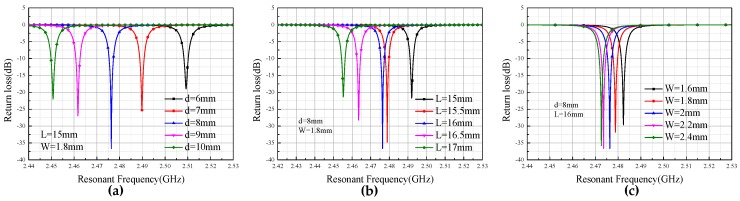
HFSS simulation results of (**a**) position *d*; (**b**) aperture length *L*; and (**c**) aperture width *W*.

Three primary parameters are present, namely, aperture length *L*, width *W*, and distance *d* between the aperture and resonator center, that determine the impedance matching between the aperture and resonator. Figure 5 shows the parametric study. The resonant frequency decreases with increased *L*, *W*, and *d*. This can be explained by the fact that the relative position of the aperture leads to a change in inductance *L_a_* and capacitance *C_a_* of the equivalent circuit and, therefore, changes the resonant frequency. The change in the relative position between the resonator and aperture also affects the coupling coefficient *K*_12_, which could also affect the impedance matching between the resonator and aperture. Finally, *L* = 16 mm, *d* = 8 mm, and *W* = 2 mm are chosen as the optimized values.

**Figure 6:**
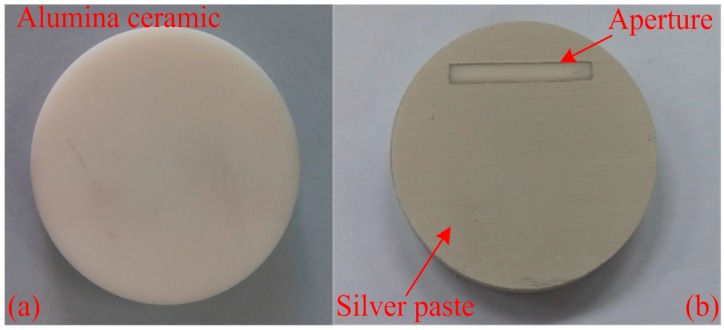
Sensor fabrication process. (**a**) Alumina ceramic; (**b**) Fabricated temperature sensor.

To form the resonator and aperture, silver paste was coated on the alumina ceramic by screen printing with a film thickness of 25 μm. The thickness and surface roughness of the metal film greatly influence the sensor signal strength [16]. Therefore, the screen-printing process should be smooth to reduce unnecessary loss. After the silver paste was coated on the ceramic, it was leveled for 10 min at room temperature, dried at 125 °C for 15 min, and sintered at 850 °C for 10 min to form a dense metal film. The fabrication process is shown in Figure 6. The broadband coplanar waveguide (CPW)-fed square slot antenna with a widened tuning stub, used as the interrogation antenna, will not be described here in detail because it has been previously discussed [17].

## 3. Temperature Measurements and Results

**Figure 7:**
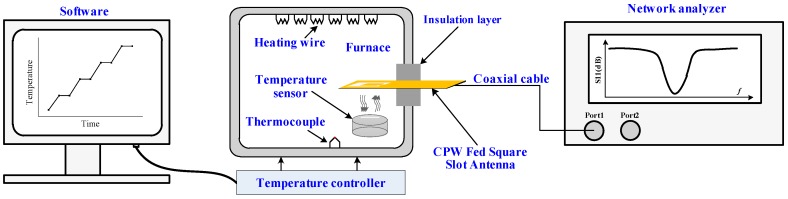
Schematic of the high-temperature measurement setup using a furnace.

To characterize the sensor high-temperature properties, a wireless measurement was set up, as shown in Figure 7. Software was utilized to set the temperature curve and send it to the temperature controller of a Nabertherm LHT 02/16 furnace (Nabertherm GmbH, Lilienthal, Germany), which was used as the heat source in these experiments. The interrogation antenna was connected to a vector network analyzer (Agilent E5061B, Agilent Technologies, Santa Clara, CA, USA) through a coaxial cable to excite the sensor and detect the backscattered spectrum signal of the sensor. The sensor was installed in the heating furnace above the interrogation antenna. We should note that the relative position between the interrogation antenna and aperture has a significant effect on the readout signal because of the directivity and polarization direction of the interrogation antenna and the aperture. The experimental results show that when the aperture was aligned with the tuning stub, maximum sensor signal strength was obtained. An insulation layer was applied to keep the temperature stable inside the furnace.

**Figure 8:**
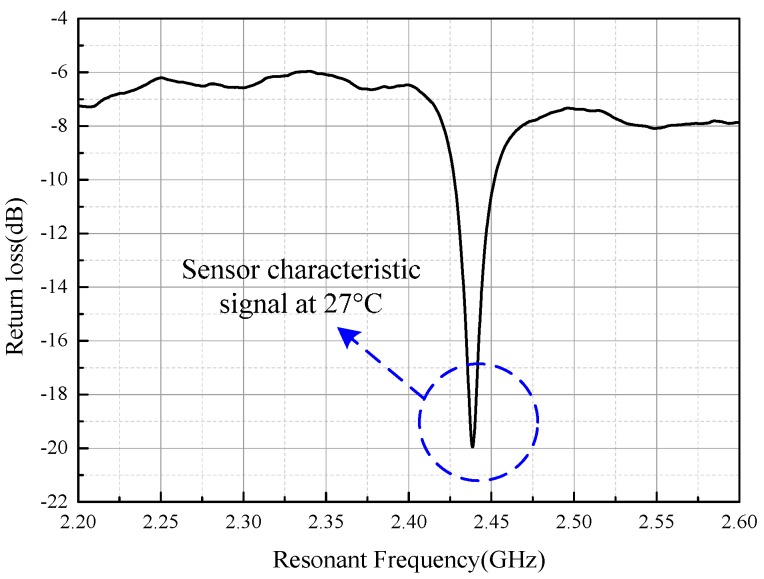
Detected original sensor response curve.

The metallic environment caused severe interference during the sensor signal extraction. In addition to the expected sensor signal backscatter, the interrogation antenna received unexpected reflections from the environment. The sensor characteristic signal was quite dim and was a small target compared with the background clutter of the signals, which rendered reading erroneous or even impossible. Figure 8 shows the sensor response in the furnace at 27 °C. The sensor characteristic signal is represented as a negative frequency as observed. Nevertheless, it still contains some ripples due to the influence of the metallic environment, which deteriorates the sensor signal at high temperature.

**Figure 9:**
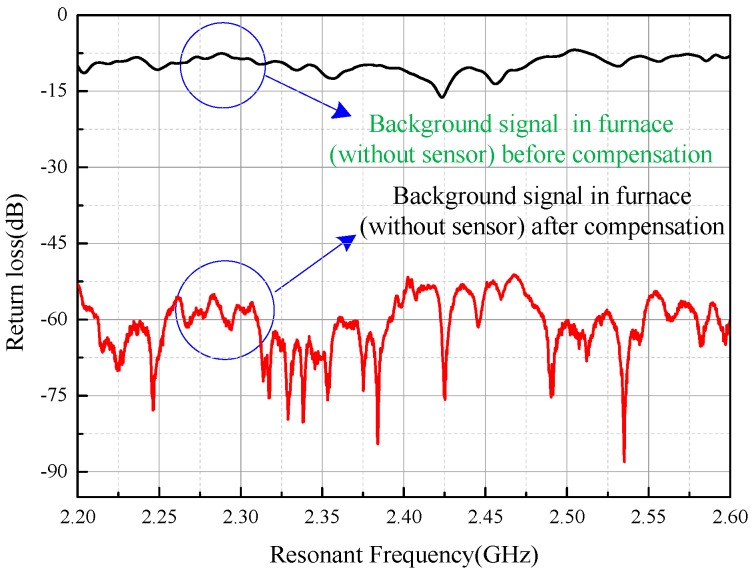
Power of the background clutter before and after frequency-domain compensation in the metal-sealed chamber.

**Figure 10:**
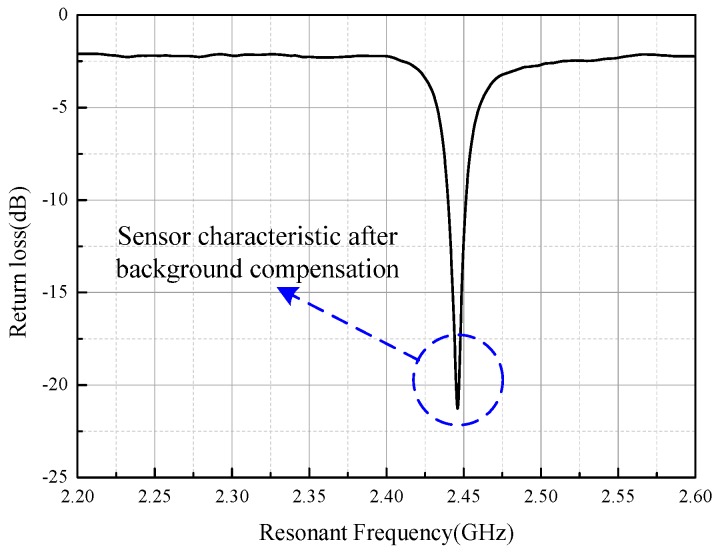
Received signal after compensation in the frequency domain.

Suppression of the background clutter was necessary to improve the signal-to-noise ratio (SNR) of the sensor signal. Frequency-domain compensation is an available option to reduce the interference of background clutter. Figure 9 shows the process of the frequency-domain compensation. The upper curve with a black line represents the power of the background clutter in the furnace (without the sensor) before compensation. The lower curve with a red line represents the background signal in the furnace (without the sensor) after compensation and is generated using the real-time background clutter data vector substrate in the upper curve, which are stored in the memory of the network analyzer beforehand. The power of the background clutter was significantly reduced from an average of −10 dB to an average of −65 dB, indicating that the frequency-domain compensation could effectively improve the SNR of the sensor signal. Figure 10 shows the signal segment after compensation with the sensor in the furnace. The curve is relatively smooth compared with that shown in Figure 8.

**Figure 11:**
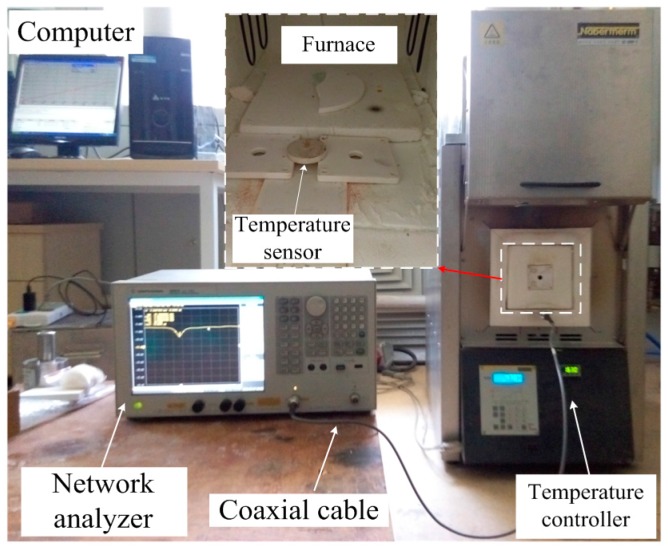
Temperature measurement setup.

**Figure 12:**
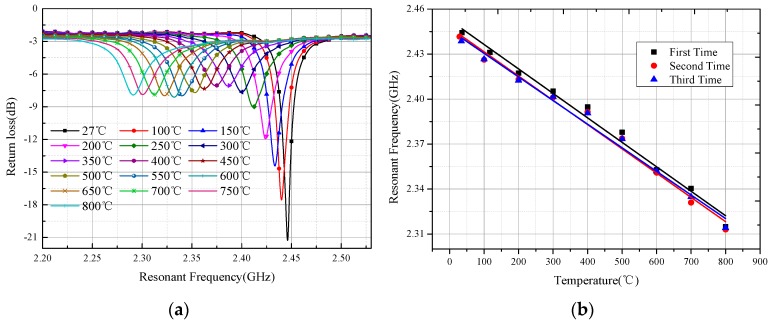
(**a**) Measured return loss curve versus frequency; (**b**) Extracted frequency versus temperature.

The temperature sensor was investigated using the setup shown in Figure 7, and the result is shown in Figure 11. The sensor resonant frequency *f*_0_ changed from 2.441 to 2.291 GHz when the temperature varied from 27 to 800 °C, leading to average absolute sensitivity s*_f_* = *∆f*_0_/*∆t* = 194 kHz/°C and average temperature coefficient τ*_f_* = *∆f*_0_/(*f*_0_*∆t) = 79.5 ppm/°C, as shown in Figure 12a. The dimensional change in the sensor was approximately 0.78% from 27 to 800 °C considering that the 99% alumina ceramic has a maximum coefficient of thermal expansion of 9.8 × 10^−6^ /°C at 800 °C. According to Equations (1) and (2), the sensor resonant-frequency change due to the thermal expansion was approximately 0.77%, which was much less than the 6.15% resonant-frequency change caused by the temperature. The thermal expansion of the sensor will decrease the dimensions of *W*, *L*, and d, which increase the resonant frequency of the sensor according to the HFSS simulation results shown in Figure 5; however, the dimension changes in *W*, *L*, and *d* are rather small and could be neglected. The measured resonant frequency at 27 °C slightly deviates from the simulated resonant frequency, which may be caused by inaccuracies in the fabrication process and the relative permittivity of the alumina ceramic. The negative peak curve gradually flattens as the temperature ranges from 27 to 300 °C, becoming stable in the temperature range from 300–800 °C, meaning that the sensor has a stably-loaded quality factor (*Q*_0_ = *f*_0_/*∆f*_3*dB*_), and is maintained at approximately 50. Repetitive experiments were conducted to verify the feasibility of this type of sensor. To ensure the same test environment, we keep the position unchanged and set the time interval between each peak to 10 min and the holding time to 20 min. By extracting the frequency peak shown in Figure 12a, a quasi-linear relationship between the temperature and frequency can be obtained. This relationship, shown in Figure 12b for the three subsequent experiments, suggests that the sensor possesses good repeatability. The slight frequency deviation among the three experiments is believed to be mainly due to the inaccuracy of the furnace temperature control and measurement system.

## 4. Conclusions

The temperature sensor presented in this paper uses alumina ceramic and silver paste for wireless sensing, and offers a great potential for harsh environment applications. The theoretical analysis, simulation, and experiments confirm the feasibility of the sensing system. The high-temperature characteristics of the sensor were investigated and discussed. The SNR of the sensor signal has improved due to the frequency-domain compensation. The extracted peak frequency was found to monotonically shift from 2.441 to 2.291 GHz when the temperature changed from 27 to 800 °C, leading to an average absolute sensitivity of 194 kHz/°C and an average temperature coefficient of 79.5 ppm/°C. The sensor was measured at 800 °C to avoid damage to the sensor because silver melts at 950 °C. However, according to the trend of the measured curve, achieving a higher temperature range is possible. Our future work will focus on optimizing the uniform fabrication of this type of sensor and improving the gain and directivity of the interrogation antenna to increase the sensing distance.
